# Pyrancoumarin derivative LP4C targeting of pyrimidine *de novo* synthesis pathway inhibits MRSA biofilm and virulence

**DOI:** 10.3389/fphar.2022.959736

**Published:** 2022-09-06

**Authors:** Yongsheng Liu, Shan Su, Moxi Yu, Dongshen Zhai, Yachen Hou, Hui Zhao, Xue Ma, Min Jia, Xiaoyan Xue, Mingkai Li

**Affiliations:** ^1^ Department of Pharmacology, Key Laboratory of Gastrointestinal Pharmacology of Chinese Materia Medical of the State Administration of Traditional Chinese Medicine, School of Pharmacy, The Fourth Military Medical University, Xi’an, China; ^2^ Precision Pharmacy and Drug Development Center, The Fourth Military Medical University, Xi’an, China

**Keywords:** *Staphylococcus aureus*, biofilm, coumarin, pyrimidine, virulence

## Abstract

*Staphylococcus aureus* poses a serious public health threat because of its multidrug resistance and biofilm formation ability. Hence, developing novel anti-biofilm agents and finding targets are needed to mitigate the proliferation of drug-resistant pathogens. In our previous study, we showed that the pyrancoumarin derivative 2-amino-4-(2,6-dichlorophenyl)-3-cyano-5-oxo-4H, 5H- pyrano [3,2c] chromene (LP4C) can destroy the biofilm of methicillin-resistant *S. aureus* (MRSA) *in vitro* and *in vivo*. Here, we further explored the possible mechanism of LP4C as a potential anti-biofilm drug. We found that LP4C inhibits the expression of enzymes involved in the *de novo* pyrimidine pathway and attenuates the virulence of MRSA USA300 strain without affecting the *agr* or *luxS* quorum sensing system. The molecular docking results indicated that LP4C forms interactions with the key amino acid residues of pyrR protein, which functions as the important regulator of bacterial pyrimidine synthesis. These findings reveal that pyrancoumarin derivative LP4C inhibits MRSA biofilm formation and targeting pyrimidine *de novo* synthesis pathway.

## Introduction

*Staphylococcus aureus* (S. au*reus*) is a major bacterial pathogen that causes a wide range of clinical infections, such as minor skin and soft tissue infections and life-threatening sepsis, by producing various toxins (Cheung et al., 2021). Owing to the extensive use of antibiotics, antibiotic-resistant bacteria, including the methicillin-resistant *S. aureus* (MRSA) strains, have emerged with increasing frequency over the past decades. Although vancomycin has been used as a drug of last resort against MRSA, it is losing potency against the clinical isolates of MRSA strains with decreased susceptibility, and vancomycin-intermediate-resistant *S. aureus* and high-level vancomycin resistance have caused considerable concerns worldwide (Otto 2012; Lakhundi and Zhang., 2018).

The evolution and spread of drug resistance depends on the antibiotic pressure exerted on susceptible bacteria and may have favored the survival of resistant strains (Bell et al., 2014). Recently, the development of anti-virulence therapeutics that inhibits the effects of bacterial toxins or block toxin production pathways has shown potential in thwarting the acquisition of antibiotic resistance (Kong et al., 2016). MRSA infections rely on the production of many toxins and enzymes. Coagulase, hemolysins, hyaluronidase, deoxyribonuclease, enterotoxins and Panton-Valentine leukocidin can destroy host tissues and enhance pathogenicity, and some of them have been detected frequently in MRSA infections (Bazzi et al., 2015; Ahmad-Mansour et al., 2021). Meanwhile, the secretion of surface proteins that initiate bacterial adherence to biotic and abiotic surfaces in hosts can induce the formation of bacterial biofilms, which are complex self-produced matrices, including polysaccharides, extracellular DNA and proteins (O'Toole et al., 2000). These biofilms can shield bacteria from antibiotics and host immune system attacks, and bacteria in the biofilm state display higher levels of persistence and resistance to stress than those in the planktonic state (Craft et al., 2019). As one of the leading causes of persistent human infections, the ability of MRSA to form biofilms is the primarily means of its antibiotic resistance and pervasiveness (Cascioferro et al., 2021). Given that most of antibiotics exert selective pressure and induce bacterial resistance and conventional antibiotics are becoming ineffective in the treatment of biofilm-forming MRSA, the mechanism underlying MRSA biofilm formation ability should be explored, and chemicals that can inhibit biofilm formation or toxin production should be identified.

Coumarin and its derivatives, which contain fused pyrone and benzene in their chemical structures, constitute an important group of compounds, and their diverse pharmacological activities have elicited considerable interest (Annunziata et al., 2020; Carneiro et al., 2021). Increasing evidence has shown that coumarin derivatives are potent antimicrobial molecules and anti-biofilm agents for a broad spectrum of microbial pathogens (Reen et al., 2018; Feng et al., 2020). Our previous studies have shown that many novel coumarin derivatives exhibit significant antibacterial activities against MRSA (Li et al., 2015; Qu et al., 2020), and a series of pyrancoumarin derivatives exert potent inhibitory effects on MRSA biofilms *in vitro* and *in vivo*. However, these derivatives have little bactericidal effects (Su et al., 2020), and the molecular mechanism by which pyrancoumarin derivatives suppress MRSA biofilms remains unclear.

In this study, we explored the mechanism of the pyrancoumarin derivative 2-amino-4-(2,6-dichlorophenyl)-3-cyano-5-oxo-4H, 5H-pyrano [3,2c] chromene (LP4C), which exhibits potent effects that inhibit biofilm formation activity against MRSA infection. The anti-MRSA biofilm formation activity is most likely through targeting bacterial pyrimidine synthesis pathway, which provides the basis and strategies for developing new antibacterial biofilm infection agents.

## Material and methods

### Bacterial strains

MRSA USA300 strain was obtained from the Chinese National Center for Surveillance of Antimicrobial Resistance (Beijing, China). An *agr*-deficient USA 300 strain (△*agr*) was obtained in our laboratory, and *PSMα-* or *PSMβ-*deficient USA 300 strain (△*PSM*α, △*PSMβ*) was provided by Professor Michael Otto, National Institute of Allergy and Infectious Diseases, National Institutes of Health, United States.

### Bacterial biofilm measurement

Bacterial biofilm was measured according to a previously described method (Su et al., 2020). MRSA USA300 was incubated in test tubes with 4 ml of tryptic soy broth (TSB) containing 2% (w/v) glucose, and then shaken at 220 rpm for 12 h at 37°C for bacterial adhesion. After 24 h of incubation at 37°C, the plate well was rinsed with 150 μl of PBS, and the attached bacteria were fixed with 150 μl of methanol and left to air dry and then stained with 150 μl of 1% (w/v) crystal violet solution for 15 min. Approximately 150 μl of 33% (v/v) glacial acetic acid was added to each stained well, and optical density (OD) was measured at 630 nm with a microplate reader (Bio-Tek ELX800, Berten, United States).

### Immunofluorescence staining

MRSA USA300 was seeded in 96-well microtiter plates with 200 μl of TSB containing 0.5% glucose. Vehicle or LP4C was added to each well to a final concentration of 50, 100 and 200 μg/ml, and an equal volume of TSB medium was added as control. The bacteria were labelled by fluorescein isothiocyanate and incubated at 37°C for 2 h. After extensive washing of the wells with PBS, the adhered cells were visualised through fluorescence microscopy (Olympus CKX41, Olympus, Japan) at 490 nm.

### Scanning electron microscopy

Then bacterial biofilm samples were washed with 0.01 M PBS and then fixed with 3% glutaraldehyde for 2 h. After 1% osmium tetroxide (OsO_4_) was used for 2 h of post-fixation, the samples were dehydrated with a graded acetone series (50, 70, 80, 90, and 95% acetone) for 15 min successively. The samples were freeze-dried, coated with gold and observed through scanning electron microscopy (HITACHI S-3400N, Hitachi, Japan).

### Silencing of *LuxS*

The silencing of genes with antisense oligonucleotides was performed as described previously (Meng et al., 2006). According to the bacterial *pyrR* gene sequence, a series of oligonucleotides was designed by using RNA structure software, and three antisense oligodeoxynucleotides were selected (Supplementary Table S2) and verified with BLAST software. MRSA USA300 in inductor receiving state was injected into a pre-cooled 0.1 mm electric shock cup (Bio-Rad, United States) and then mixed with oligodeoxynucleotides. The settings of the electroporation instrument were as follows: 25 μF, 900 V and 200 Ω. After electric shock, the pre-heated medium at 37°C was quickly added and resuscitated at 37°C for 1 h, and the resuscitated bacterial solution was diluted to the appropriated concentration.

### Molecular docking

No available PyrR protein structure model of *S. aureus* was found in PDB database, and thus we used the SWISS-MODEL to build a homologous model according to the existing PyrR protein structure model (PDBID: 4P83). The 3D structure model of LP4C was constructed using ChemBio3D software (Version:14.0.0.117). AutoDock Tools (Version:1.5.6) was used to dock LP4c and the active site of the homologous PyrR protein model. The energy of the LP4C molecule was minimised using ChemBio3D and used as an input for AutoDock Tools. The docking algorithm provided by AutoDock Tools was used in determining the best docked conformation between LP4C and PryR protein. The conformations with the most favorable free binding energy were selected for analyzing the interactions between the LP4C and protein by Discovery Studio 2019 (Version:19.1.0.18287).

### Real-time PCR

Total RNA was isolated using a bacterial RNA isolation kit (Tiangen, Beijing, China) according to the manufacturer’s protocol. PCR primers for each tested gene are presented in Supplementary Table S1. The total RNA was reverse-transcribed into cDNA using the PrimeScript RT reagent kit with gDNA Eraser. For real-time PCR (RT-PCR), 1 μl of cDNA was used as the template, and the reaction was catalyzed with the SYBR Premix Ex Taq (TaKaRa, Japan). RT-PCR amplification was performed using a multiplex quantitative PCR system (Stratagene Mx3005P, Agilent, United States), and the amplification condition of the RT-PCR was pre-denaturation at 95°C for 30 s, followed by 40 cycles of denaturation at 95°C for 10 s and annealing at 60°C for 30 s. Detection of the fluorescent product was carried out at the end of the 72°C elongation incubation. The relative expression levels of the target genes was calculated by the 2^-△△Ct^ method.

### Transcriptome analysis

MRSA USA300 cell pellets were harvested through centrifugation and subjected to RNA extraction. The cDNA was prepared from the total RNA through reverse transcription, and the sequencing libraries were generated using a gene-sequencing system (HiSeq 2000, Illumina, United States). The fragments per kilobase million measurements were used in comparing the expression of genes between the control and LP4C treatment groups. In transcriptomic data analysis, the original Affymetrix data was pre-processed using an oligo R package and normalised and log-transformed using a multi-array average (RMA) method. Log2 (fold change) of ≥1 and *Padj* value of ≤0.05 were considered significant differences in gene expression. Kyoto Encyclopedia of Genes and Genomes (KEGG) and Gene Ontology (GO) analyses were performed for the mapping of involved pathways.

### Statistical analysis

Statistical analyses were performed using Prism version 8.1 (Graph Pad, United States), and data were expressed as mean ± SD, and the mean was obtained from at least three replicates. Statistical significance was determined using one-way ANOVA (analysis of variance), two-way ANOVA and Kaplan-Meier survival analysis. A probability value of <0.05 was considered indicative of statistical significance.

## Results

### Effect of LP4C on the extracellular biofilm matrix of MRSA

**FIGURE 1:**
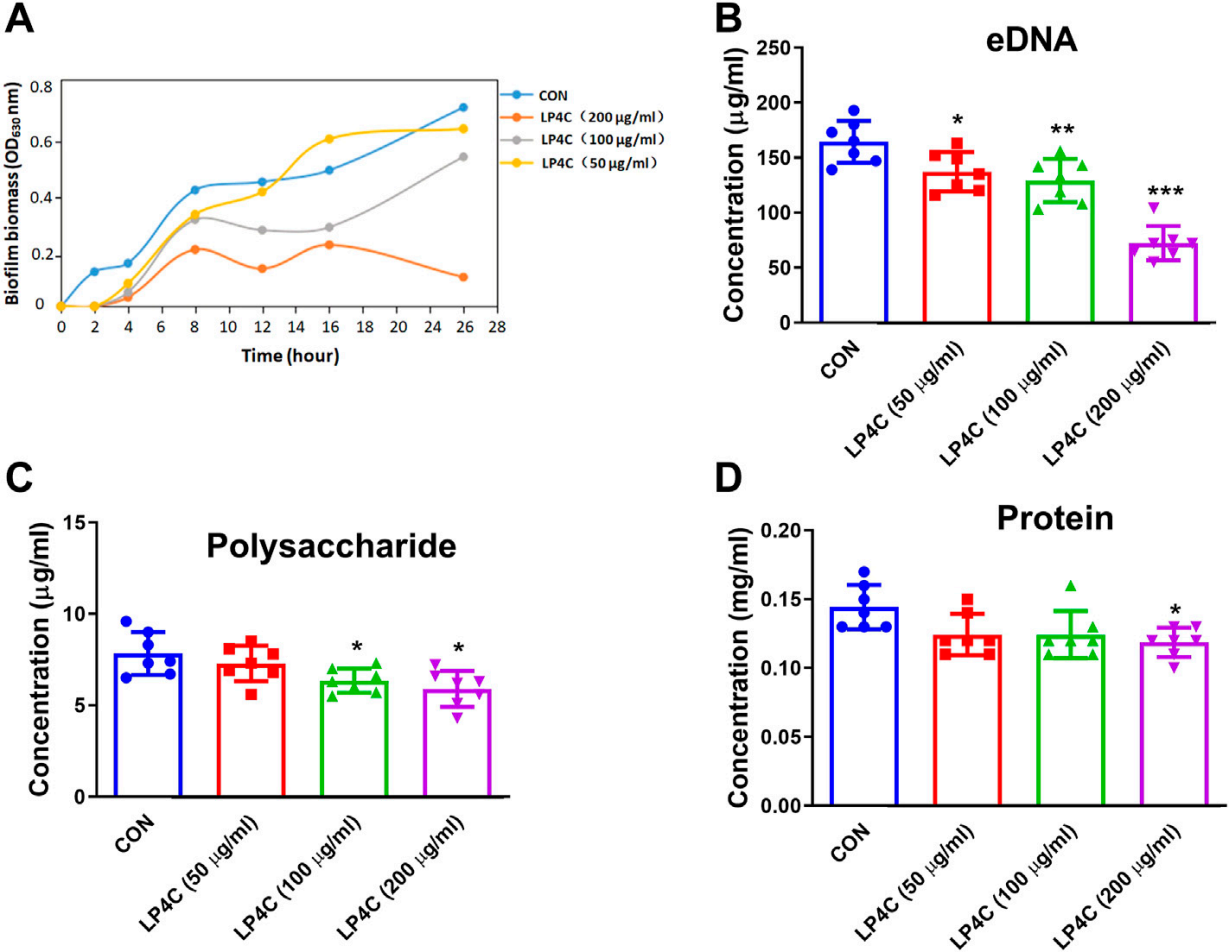
LP4C inhibits the extracellular biofilm matrix of MRSA. The inhibitory activity of LP4C (50, 100 and 200 μg/ml) to the dynamic of MRSA USA 300 biofilm biomass **(A)**. The effect of LP4C (50, 100 and 200 μg/ml) to the concentration of eDNA **(B)**, polysaccharide **(C)** and protein **(D)**, *p < 0.05, **p < 0.01, ***p < 0.001 vs*.* control (CON), n = 7.

Extracellular polymeric substance (EPS) is pivotal in the protection of biofilm inhabitants against mechanical and chemical challenges, and thus the inhibition or dispersal of the EPS matrix in biofilm-associated infection is critical to the development of novel therapies (Hou et al., 2018). Firstly, we observed the inhibitory effects of LP4C on MRSA biofilm biomass. The result showed that LP4C (100 and 200 μg/ml) inhibited the formation of biofilm biomass of MRSA USA 300 significantly, especially within 24 h (Figure 1A). Given that the bacterial biofilm is a matrix of polysaccharides, extracellular DNA (eDNA) and protein, we measured the effect of LP4C on these EPSs. Compared with the vehicle control, LP4C (50, 100 and 200 μg/ml) considerably reduced the level of eDNA (Figure 1B), and the treatment inhibited extracellular polysaccharide or protein at 100 and 200 μg/ml concentrations (Figures 1C,D). These results indicated that LP4C can inhibit the production or secretion of EPS, particularly the concentration of eDNA in MRSA biofilms.

### LP4C inhibits the formation of MRSA biofilms through the bacterial pyrimidine biosynthesis pathway

**FIGURE 2:**
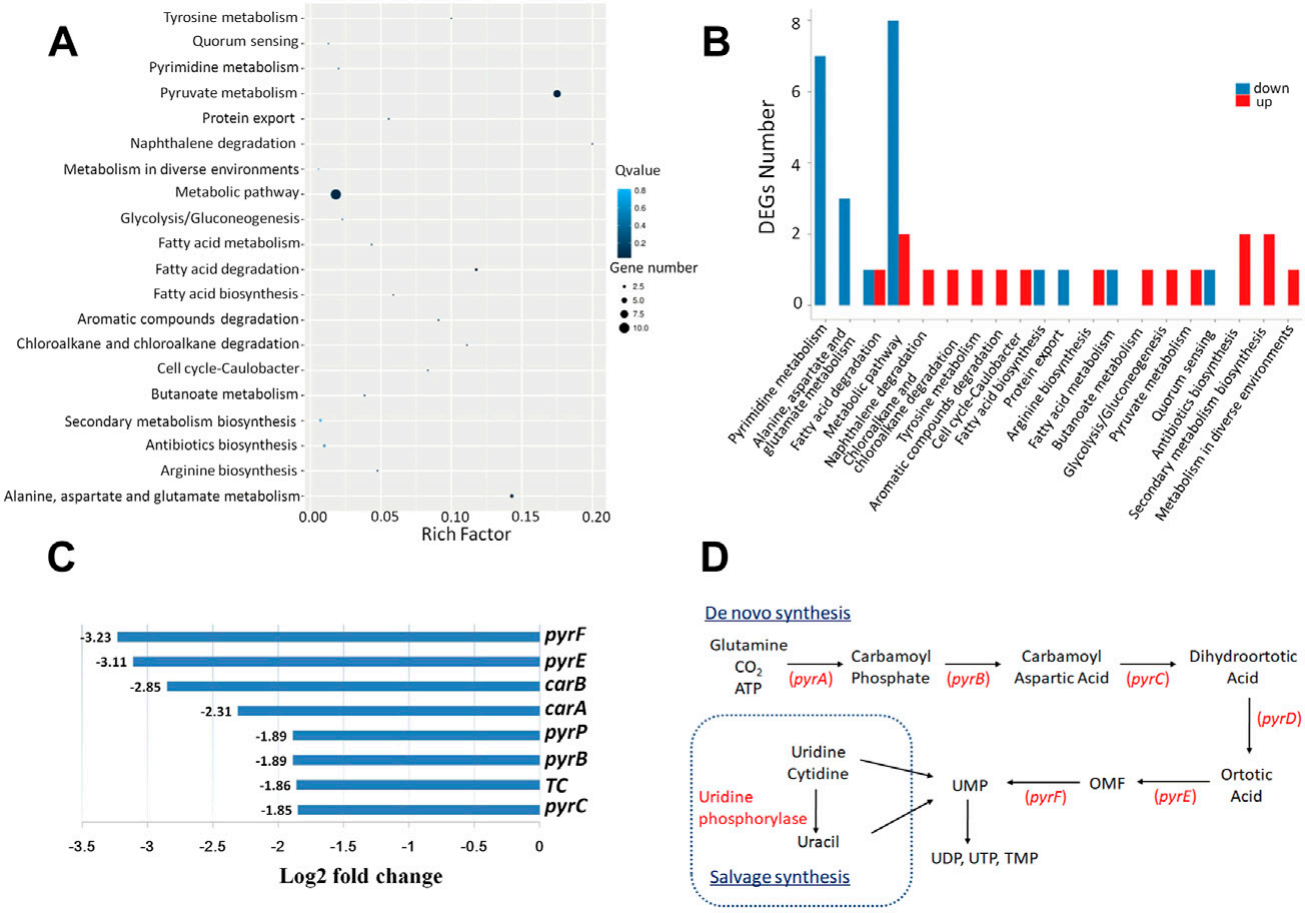
LP4C downregulates the expression of genes involved in the pyrimidine *de novo* synthesis pathway of MRSA. Top 20 statistics of pathway enrichment after compound LP4C (100 μg/ml) treatment to MRSA USA 300 **(A)**. The DEGs number of most enriched pathway of MRSA USA 300 treated by compound LP4C **(B)**. The log2 fold change value of top down-regulated expressed genes **(C)**. The illustration of *de novo* salvage bacterial pyrimidine synthesis pathway **(D)**.

To explore the possible mechanisms of LP4C, we performed RNA sequencing and transcriptome analysis on MRSA 300 in the presence or absence of LP4C. The result revealed that LP4C modulated the expression of 25 genes (nine upregulated and 16 downregulated genes). The top 20 statistics of pathway enrichment data showed that LP4C (100 μg/ml) modulated the expression of genes mainly involved in pyrimidine metabolic pathways. This feature was also indicated by the number of DEGs of the most enriched pathway of MRSA USA 300 (Figures 2A,B). In the most differentially down-regulated expressed genes, the log_2_fold change values of *pyrF*, *pyre, carB, carA* were −3.23, −3.11, −2.85 and −2.31, respectively, and the values of *pyrP, pyrB* and *pyrC* were −1.89, −1.89, and −1.85, respectively (Figure 2C). These genes encode proteins, such as orotidine-5′-phosphate decarboxylase, orotate phosphoribosyltransferase, carbamoyl-phosphate synthase, uracil permease and aspartate carbamoyltransferase, which are involved in the *de novo* synthesis pathway of bacterial pyrimidine (Figure 2D). The transcriptomic profiling displayed that LP4C exert inhibitory effects by reducing the expression of key enzymes involved in the MRSA pyrimidine synthesis pathway.

### Inhibitory activity of LP4C is independent of agr/AI-2 quorum sensing system

**FIGURE 3:**
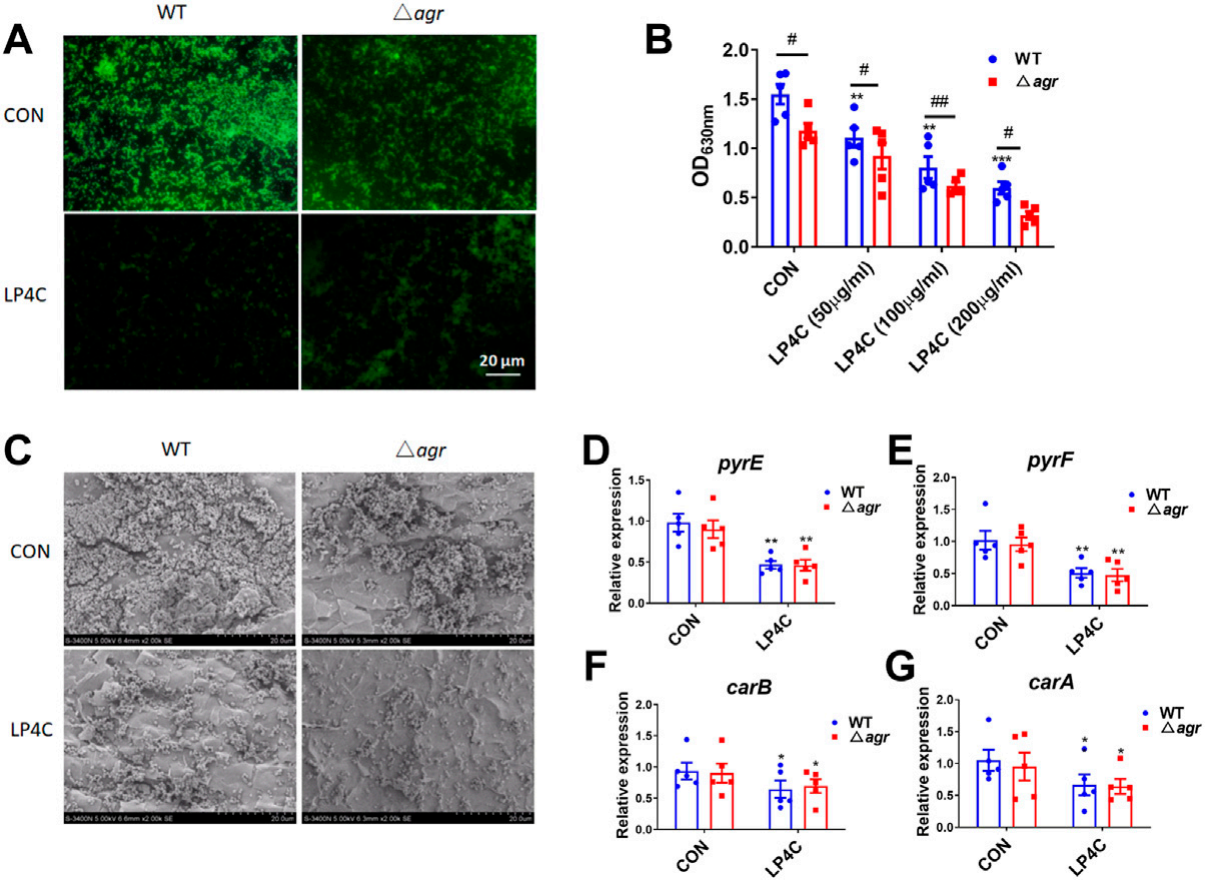
Agr QSS is not involved in the inhibitory activity of LP4C. Immunofluorescence staining of wild type (WT) and *agr* deficiency (∆agr) MRSA USA300 in control (CON) or LP4C treatment **(A)**. Morphological observation of biofilm structure and bacteria number of WT or ∆agr MRSA USA300 under the SEM after LP4C treatment **(B)**. The effect of LP4C (50, 100 and 200 μg/ml) to the formation of WT and ∆agr MRSA USA300 by crystal violet staining, **p < 0.01, ***p < 0.001 vs*.* control (CON), ^#^ *p* < 0.05, ^##^ *p* < 0.01 vs*.* ∆agr group, n = 5 **(C)**. The effect of LP4C (100 μg/ml) to the gene expression of WT and ∆agr MRSA USA300, *p < 0.05, **p < 0.01 vs*.* control (CON), n = 5 **(D–G)**.

Quorum sensing system (QSS) is commonly employed in bacteria to regulate the biofilm formation and virulence factors expression. Several types of QSS such as the accessory gene regulator (agr) in Gram-positive bacteria play a crucial role on *Staphylococcus* pathogenesis, and many inhibitors can block QSS-dependent virulence gene expression and biofilm formation without detectable bactericidal activity (Brackman and Coenye, 2015; Tan et al., 2018). Considering that LP4C has characteristics similar to the QSS inhibitor, we determined whether QSS mediates the anti-biofilm activity of LP4C. Given that agr QSS can promote the production of many virulence factors and MRSA biofilm formation, we then observed the biofilm formation of *agr*-deficient (∆agr) MRSA USA300. The results showed that ∆agr MRSA had impaired formation ability compared with the wild type (WT) MRSA, and LP4C (100 μg/ml) treatment further inhibited biofilm formation (Figure 3A). Compared to the WT MRSA USA300, the structure of MRSA biofilm was much lower in the ∆*agr* MRSA USA300 under the scanning electron microscope, and LP4C (100 μg/ml) inhibited biofilm formation (Figure 3B). Crystal violet staining showed that LP4C (50, 100 and 200 μg/ml) not only inhibited the WT MRSA USA300 biofilm formation in the concentration-dependent model but also inhibited ∆agr MRSA USA300 biofilm formation (Figure 3C). Consistently, LP4C (100 μg/ml) significantly inhibited the expression of *pyrE*, *pyrF*, *carB* and *carA* genes in WT and ∆agr MRSA USA300 (Figures 3D–G). Our results confirmed the role of agr QSS in MRSA biofilm formation and virulence factor expression in MRSA. LP4C further inhibited biofilm formation in the agr-deficient MRSA strain. This result indicated that agr QSS is unlikely involved in the anti-biofilm activity of LP4C.

LuxS/AI-2 is another kind of important quorum sensing system of various strains, including *S. aureus*. It detects autoinducers, regulates the transcription of target genes and affects growth characteristics, biofilm formation and virulence (Le and Otto., 2015). To determine whether LuxS/AI-2 QSS controls the expression of key enzymes involved in bacterial pyrimidine synthesis pathway and mediates the activity of LP4C, we designed and synthesised three antisense oligonucletides to silence the *luxS* gene. The results showed that oligonucletide 1 reduced the expression of *luxS* gene by about 40% in MRSA USA300 (Supplementary Figure S1A). The parameter spectra of oligonucletide 1 is shown in Supplementary Figure S1B. The expression levels of *pyrE*, *pyrF*, *carA* and *carB* were not regulated on the MRSA USA300 pre-treated with oligonucletide 1 in contrast to those in the control MRSA USA300, and LP4C attenuated the expression of *pyrE*, *pyrF*, *carA* and *carB* on the luxS-silencing MRSA (Supplementary Figures S1C–F). These data indicated that the inhibitory activity of LP4C is independent of agr QSS or LuxS/AI-2 QSS.

### Molecular recognition of PyrR protein by LP4C

The genes involved in pyrimidine ribonucleotide biosynthesis were involved with most donw-regulated pathway. *pyr* genes such as *pyrP*, *pyrB*, *pyrC*, *carA*, *carB*, *pyrF* and *pyrE*, were located on the same operon and transcribed from a single promoter regulated by pyrR-mediated transcriptional attenuation (Turnbough and Switzer., 2008; Buvelot et al., 2021). We investigated whether LP4C downregulates *pyr* genes by targeting the PyrR protein. Owing to the lack of available *S. aureus* PyrR nucleotide sequence information, we firstly selected the best matched degree template as the molecular docking model. The matching degree between the template and target amino acid sequence was 53.76%. The Global Model Quality Estimation of the model was 0.83. These data indicated the consistency between the model and template structure, and the credibility was more than 95% (Supplementary Figure S2).

**FIGURE 4:**
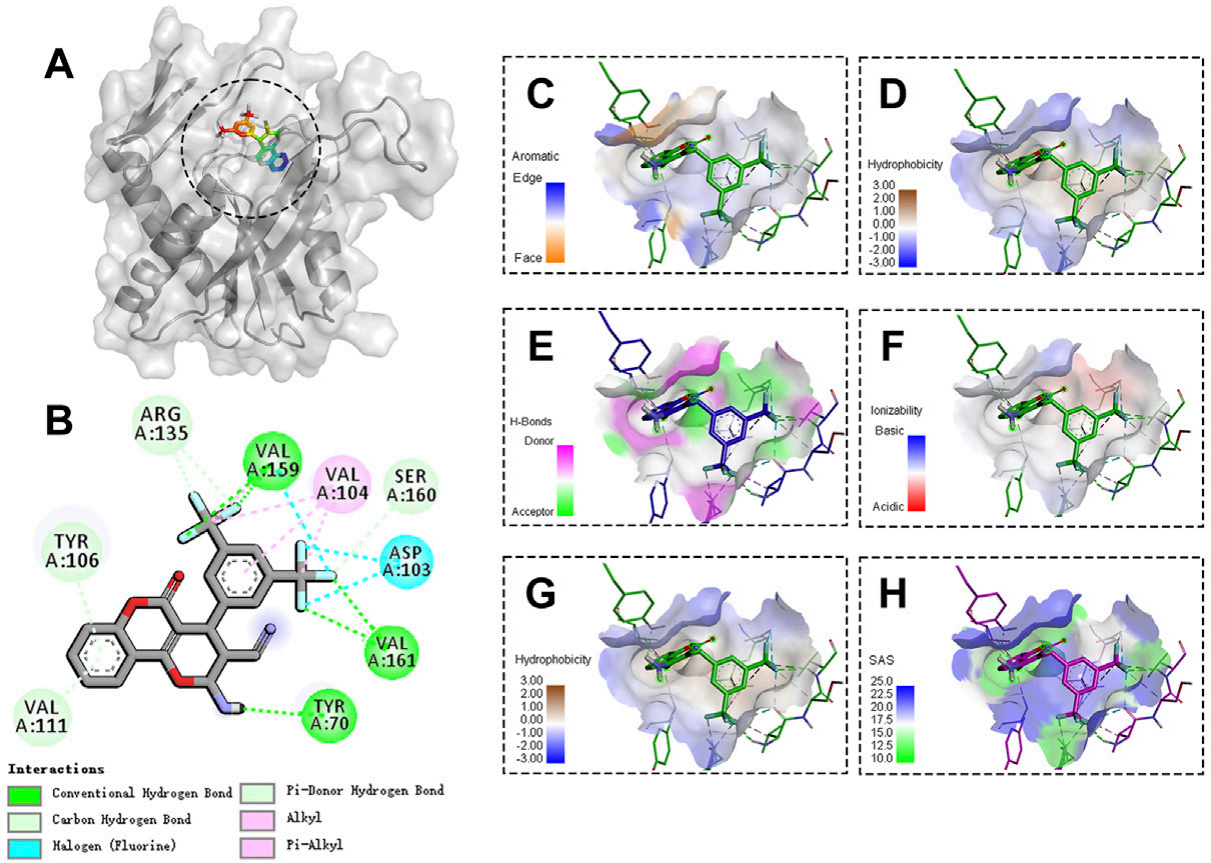
Binding site and model of LP4C with PyrR. Overview of the docked pose between the LP4C and binding pocket to the active site of PyrR**(A)**. The close view of the interaction between LP4C and the active site of PyrR **(B)**. The analyzed result of the aromatic ring edges or faces **(C)**, hydrophobicity **(D)**, hydrogen bond **(E)** and ionizability **(F)**. The analyzed result of the atomic charge, values less than -0.1 are mapped in red, and values larger than +0.1 are mapped in blue **(G)**. The analyzed result of the solvent accessibility surface, small values (green) correspond to buried residues, whereas large values (blue) correspond to exposed residues **(H)**.

The molecular docking results showed that LP4C interacted with the key amino acid residues of PyrR protein at its active site, and the molecular docking result was −7.59 kcal/mol and revealed several molecular interactions responsible for the observed affinity: 1) carbon–hydrogen bond interactions between the amino group or fluorine atom with A70, A159 and A161 residues in the PyrR protein; 2) Pi-donor hydrogen and carbon hydrogen bond interactions between the benzopyrone ring or fluorine atom and A106, A111, A135 and A160 residues; 3) alkyl and pi-alkyl interactions between the benzopyrone ring and methyl group with A104 residue; and 4) halogen interaction between the fluorine atom and A103 residue. In addition, the binding surface model of LP4C and PyrR protein was analysed, including the aromatic ring edges or faces, hydrophobicity, hydrogen bond, ionizability, atomic charge and solvent accessibility surface (Figure 4).

### Pyrimidine restores the LP4C impaired biofilm formation ability of MRSA

**FIGURE 5:**
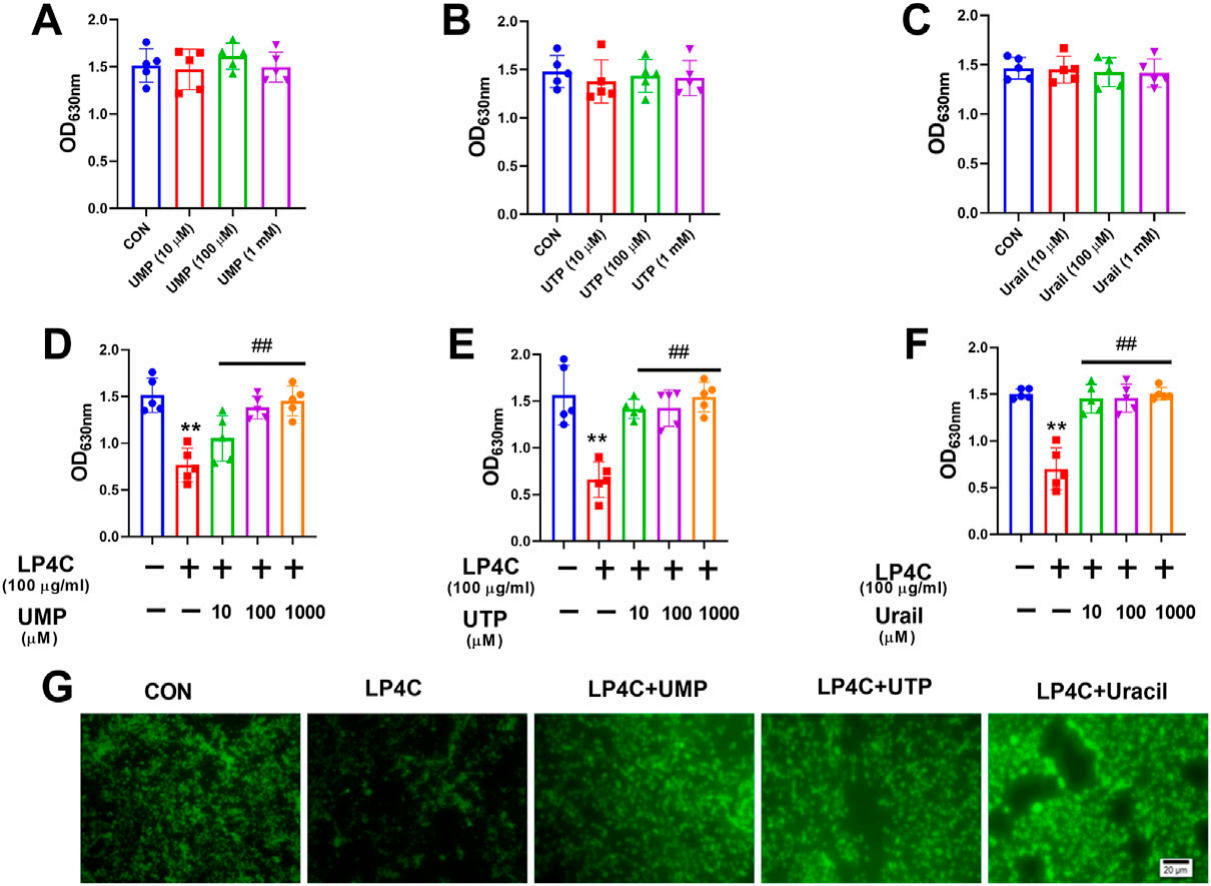
Pyrimidine restores LP4C impaired biofilm formation ability of MRSA. The biofilm formation of MRSA USA300 was observed after supplementation of UMP **(A)**, UTP **(B)** or uracil **(C)** by crystal violet staining. The UMP **(D)**, UTP **(E)** or uracil **(F)** restored the impaired biofilm formation of MRSA USA300 after the LP4C treatment, **p < 0.01 vs*.* untreated control group, ^##^ *p* < 0.01 vs*.* LP4C treated group, n = 5. The effect of UMP, UTP or uracil supplementation to the adherence capability of MRSA USA300 after LP4C treatment **(G)**.

The biosynthesis of bacterial pyrimidine involves *de novo* and salvage pathways. The former uses glutamine, ATP and bicarbonate for uridine monophosphate (UMP) and uridine-5′-triphosphate (UTP) synthesis, whereas the latter uses extracellular uracil to synthesise UMP (Figure 2D). Given that LP4C inhibited the production of pyrimidine and biofilm formation of MRSA USA300, we determined whether pyrimidine can restore the biofilm formation ability impaired by LP4C. Firstly, the culture medium was supplemented with UMP and UTP (10 μM, 100 μM, and 10 mM) from the *de novo* pathway or uracil (10 μM, 100 μM, and 10 mM) from the salvage pathway. No significant difference biofilm formation was found between MRSAUSA300 and the control (Figures 5A–C). This treatment might be saturated for unimpaired MRSA, and thus we tested the effect of the compensation of pyrimidine on LP4C-treated MRSA USA300. The results showed that supplementation of pyrimidine with UMP, UTP and uracil (10 μM, 100 μM, 10 mM) restored biofilm formation impaired by LP4C in MRSA USA300 (Figures 5D–F). Consistently, immunofluorescence staining showed that UMP, UTP and uracil (100 μM) compensated the adherence capability of MRSA USA300 after the 100 μg/ml LP4C treatment (Figure 5G).

### LP4C inhibits the expression of biofilm-associated genes in MRSA

**FIGURE 6:**
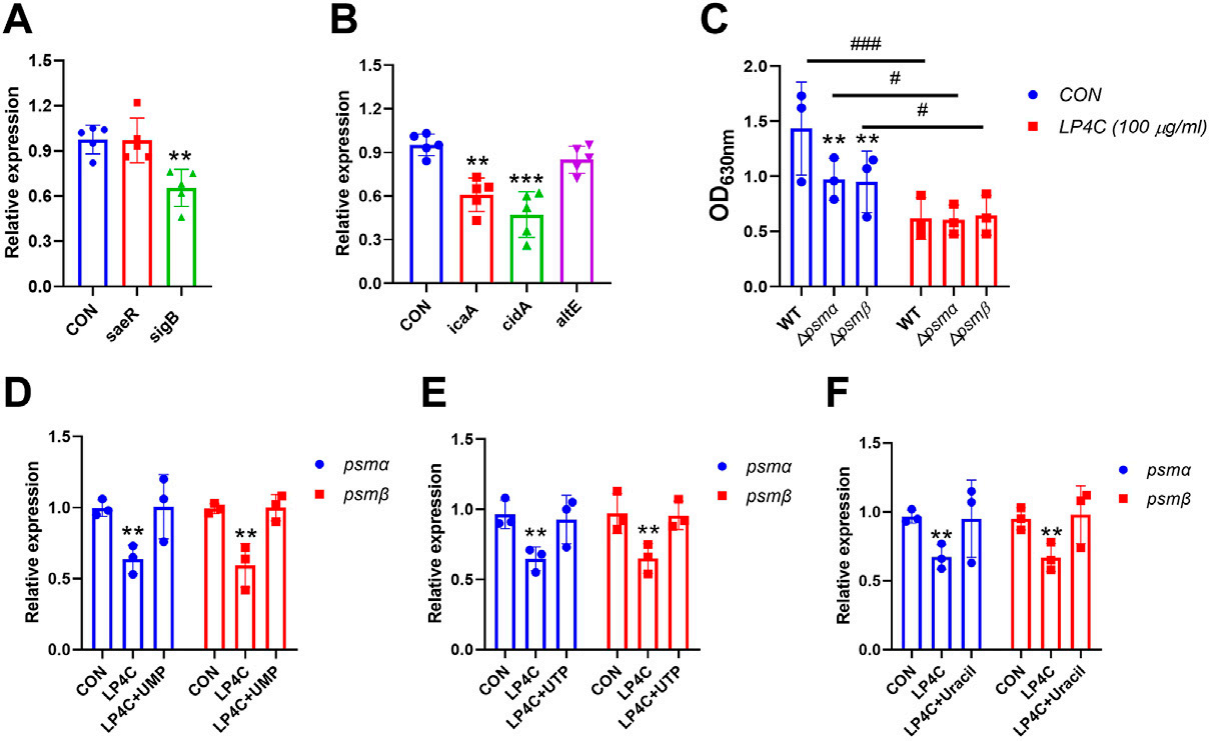
The effect of LP4C to the expression of MRSA biofilm formation associated genes. The effect of LP4C (100 μg/ml) to the expression of *sarR* and *sigB* in MRSA USA 300, **p < 0.01 vs*.* control (CON), n = 5 **(A)**. The effect of LP4C (100 μg/ml) to the expression of *icaA*, *cidA* and *altE,* data were normalized to levels of *gyrB* and calculated as fold changes, **p < 0.01, ***p < 0.001 vs*.* control (CON), n = 5 **(B)**. The impaired biofilm formation capability of mutated *psm*α (△*psm*α) or *psm*β (△*psm*β) MRSA USA300, **p < 0.01 vs*.* wild type (WT) MRSA USA300, ^#^ *p* < 0.05, ^###^ *p* < 0.0001 vs*.* control (CON), n = 3 **(C)**. The relative expression levels of *psmα* or *psmβ* of MRSA USA 300 after treatment of LP4C (100 μg/ml) with or without 100 μM UMP **(D)**, UTP **(E)** or Uracil **(F)**, **p < 0.01 vs*.* control (CON), n = 3.

Many genes are involved in biofilm development in *S. aureus*, such as *icaA*, which is required for the colonisation of hosts and *cidA* expression, which is associated with cell lysis and eDNA release during planktonic growth (Rice et al., 2007; Harapanahalli et al., 2015). To test the effect of LP4C on the expression of biofilm-associated genes in MRSA, we examined differences in the expression of important genes, including *icaA*, *cidA*, *psmα*, *psmβ*, *sigB*, *atlA* and *aseR*, between the control and LP4C-treated MRSA USA300. The results showed that LP4C significantly altered the expression of not only biofilm-regulatory genes, such as *sigB* and *saeR* (Figure 6A), but also genes related to bacterial adhesion and biomass production, such as *icaA* and *cidA* (Figure 6B). The phenol-soluble modulin (PSM) peptide family performs various virulence activities and mediates the formation of the amyloid fibrils with diverse architecture. In *psmα-* or *psmβ-*mutated MRSA USA300, biofilm formation capability was reduced by about 30%, and LP4C attenuated biofilm development (Figure 6C). Finally, supplementation with 100 μM UMP, UTP and uracil reversed the inhibitory activity of LP4C on the expression of *psmα* and *psmβ* (Figures 6D–F). Collectively, these results suggested that LP4C combats MRSA biofilm infection by inhibiting the expression of biofilm-associated factors.

## Disscusion

Currently, coumarin derivatives have received considerable interest because of their potent antimicrobial properties and are emerging as promising candidates for antimicrobial drug development (Hu et al., 2018). Rather than being active bactericidal molecules, coumarin derivatives play roles as antibacterial biofilm agents on a number of microbial pathogens, including *S. aureus*, but the precise mechanism of their anti-biofilm actions has not been reported (Das et al., 2018; Thakur et al., 2020). We firstly demonstrated that pyrancoumarin derivative LP4C inhibits MRSA biofilm formation through bacterial *de novo* pyrimidine synthesis pathway.

Notably, pyrimidine nucleotides and their derivatives, such as UTP and UMP, are essential to all living organisms. Interestingly, our previous results showed that the minimal inhibitory concentration of LP4C was higher than 256 μg/ml (Su et al., 2020), indicating that this compound has no obvious bactericidal activity against MRSA USA300. The underlying reason was the bacterial pyrimidine salvage synthesis pathway, which is usually stimulated upon the inhibition of *de novo* pyrimidine synthesis (Garavito et al., 2015). In a previous report, *pyrE* mutant colonises host poorly in competitive infection with isogenic wild-type *Salmonella typhimurium*, and the ability of this mutant was restored by providing a copy of *pyrE* in trans. This finding suggested that genes encoding proteins involved in pyrimidine *de novo* synthesis pathway play important role in the bacterial adherence and biofilm formation (Garavaglia et al., 2012; Yang et al., 2017). Furthermore, the enzymatic steps of the pyrimidine nucleotide biosynthetic pathway are the same in all bacteria, subsets of enzymes probably vary in the pyrimidine pathways of different organisms and the presence or absence of specific pyrimidine enzymes in different species evolved depending on the availability and accessibility of nutrients. These marked differences render the pyrimidine synthesis pathway a potential drug target (West, 2014; Chunduru and West, 2018).

PyrR is a protein that regulates the expression of genes and operons of pyrimidine nucleotide biosynthesis by binding to specific sequences on *pyr* mRNA in many Gram-positive bacteria (Fields and Switzer, 2007; Turnbough and Switzer, 2008). Given that the sequences of *pyrR* genes are well conserved, we performed molecular docking to observe the possible binding of LP4C with PyrR protein. The results revealed that LP4C bound with the probable target sequence-binding sites of PyrR. However, the limitation of this research is lack of a purified PyrR and precise affinity measurement between LP4C and PyrR protein.

Given that bacterial QSS is a cell–cell communication mechanism that is closely interconnected with bacterial biofilm formation and virulence factor production (Butrico and Cassat, 2020; Schilcher and Horswill, 2020), we examined the possible role of QSS in anti-biofilm activity of LP4C by using *agr-*deficient or *luxS-*silencing MRSA USA300. The *agr-*deficient strains exhibited impaired adherence and biofilm formation ability in contrast to the isogenic wild type strain, consistent with the role of agr QSS (Dotto et al., 2021). LP4C attenuated biofilm formation in the *agr-*deficient strains (Figure 3). Meanwhile, in the *luxS-*silencing strains, the expression of genes involved in the pyrimdine synthesis pathway was not altered, and LP4C reduced their levels significantly (Figure 4). Although the function of AI-2 QSS in many bacteria and the physiological role of LuxS remain controversial (Doherty et al., 2006), our finding confirmed that luxS does not mediate the pyrimidine synthesis pathway gene expression inhibited by LP4C. Although the suppression of the pyrimidine synthesis pathway can explain some of the activity, the molecular mechanism by which LP4C affects QSS and bacterial biofilm remains unclear.

The anti-biofilm activities of coumarin compounds derived from plant extracts haves been extensively studied, and the structure–function analysis revealed that naturally produced parent molecular coumarin compounds, such as esculetin and warfarin, usually possess low activities (Durig et al., 2010; Ojima et al., 2016). In this study, the pyrano-decorated coumarin derivative LP4C exhibited potent anti-biofilm activity in MRSA. However, the role of pyrancoumarin in biofilm formation inhibition needs to be confirmed in a broad range of human bacterial pathogens.

In summary, the present study suggests that pyrimidine *de novo* pathway is an attractive target for the development of novel antimycobacterial agents, and we highlighted the pyrancoumarin compound LP4C as a promising therapeutic agent for eradicating staphylococcal biofilms. This study will open a new area of research for the investigation and optimisation of coumarin in combination with traditional antibiotics and prevention of infections by drug-resistant bacteria.

## Data Availability

The datasets presented in this study can be found in online repositories. The names of the repository/repositories and accession number(s) can be found below: https://www.ebi.ac.uk/ena/; PRJEB54101.

## Supplementary material

Supplementary Figure S1 The effect of LP4C on the pyrimidine synthesis pathways related gene expression on luxS silencing MRSA. The blocking effect of three designed antisense oligonucleotides on luxS gene expression of MRSA USA 300, **p 0.01 vs control (CON), n = 5 **(A)**. Parameter spectra of the antisense oligonucleotide 1 **(B)**. The relative expression levels of pyrE **(C)**, pyrF **(D)**, carA **(E)** and carB (F) of MRSA USA 300 after oligonucleotide 1 or combination with 100 μg/ml LP4C treatment, *p 0.05, **p 0.01 vs. control (CON), n = 5.

Supplementary Figure S2 Chemical Structure of LP4C and homology modeling of PyrR protein. The chemical structure of pyrancoumarin cderivative LP4C **(A)**. The homology modeling S. aureus structure of PyrR protein, the part of the model with low credibility of the Quaternary Structure was shown in orange, and the credibility near the active site was shown in blue **(B)**. Most of the predicted local similarity to target shown by Local Quality Estimate is greater than 0.6, and the GMQE was 0.81 **(C)**. Consensus-based distance constraint (DisCo) score was 0.77 ±0.05, and the credibility was more than 95% **(D)**.
