# Supplementary figures and images for: Pyrancoumarin derivative LP4C targeting of pyrimidine de novo synthesis pathway inhibits MRSA biofilm and virulence
Source: Front Pharmacol. 2022 Sep 6;13:959736. doi: 10.3389/fphar.2022.959736 (PMC9486200; doi:10.3389/fphar.2022.959736)

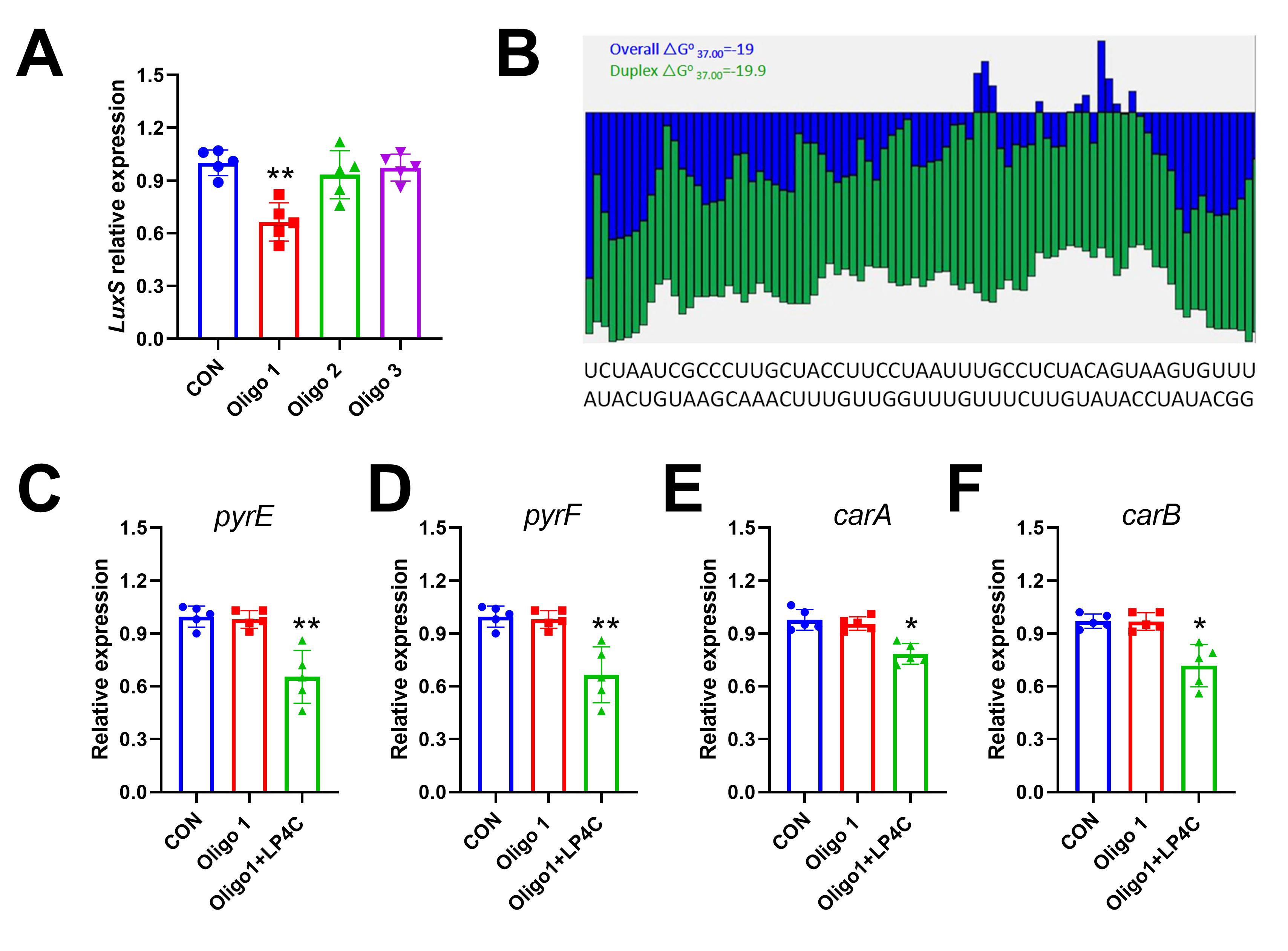

Supplement: Supplementary file 1 [file Image1.JPEG]

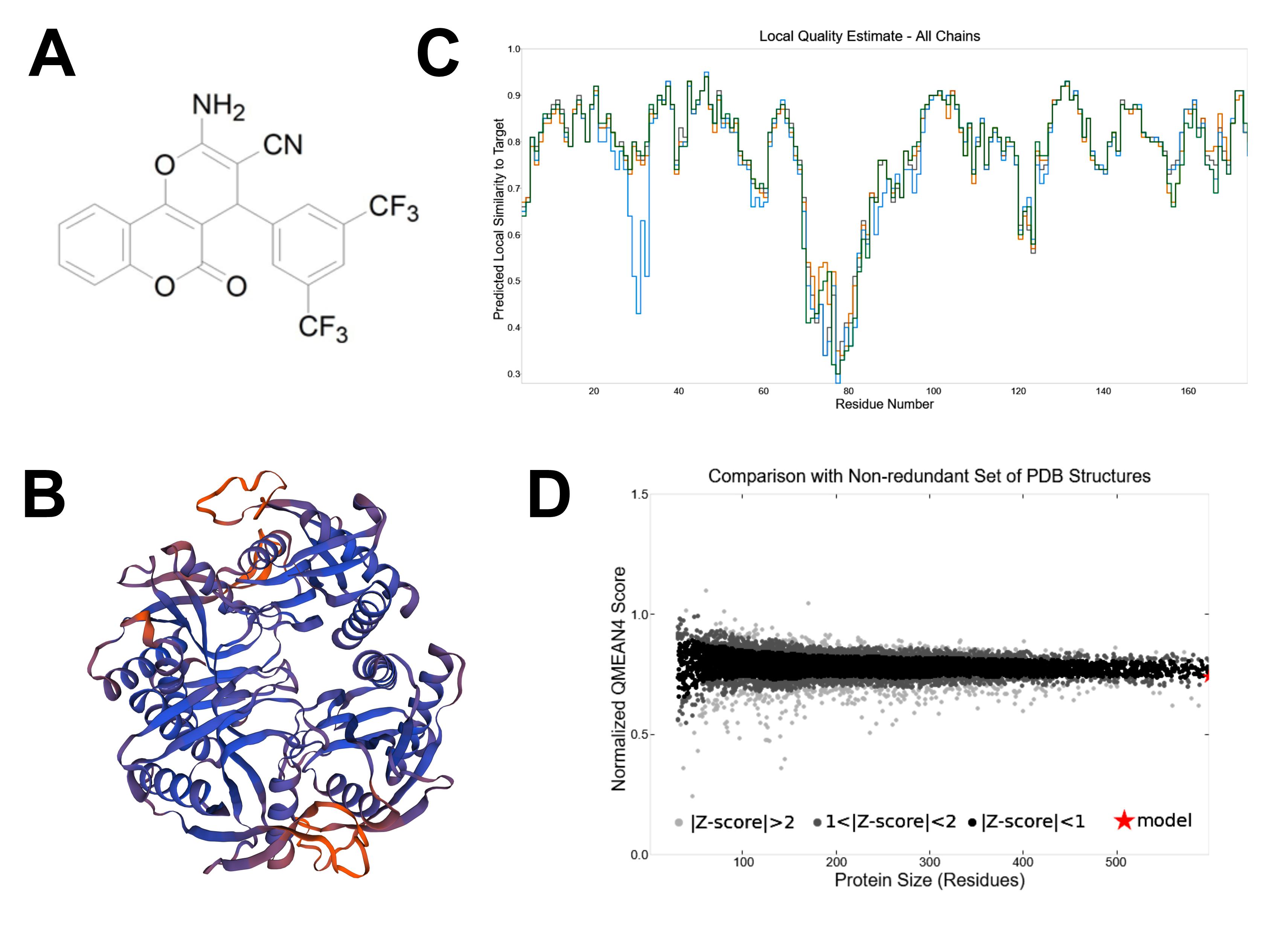

Supplement: Supplementary file 2 [file Image2.JPEG]
