# Supplementary material for: Pyrancoumarin derivative LP4C targeting of pyrimidine de novo synthesis pathway inhibits MRSA biofilm and virulence
Source: Front Pharmacol. 2022 Sep 6;13:959736. doi: 10.3389/fphar.2022.959736 (PMC9486200; doi:10.3389/fphar.2022.959736)
Supplement: Supplementary file 3 [file DataSheet1.docx]

**Supplementary** **Table 1 Primers of RT-PCR**

| Gene |  | Primer sequence (5’-3’) |
| --- | --- | --- |
| *carA* | Forward | GACAGCGGCGTGGTGATGATC |
|  | Reverse | CTTGTGAGTGGCTCGCAGGTTG |
| *carB* | Forward | CGTGCCGTTCGTCTCCAAGTG |
|  | Reverse | GCGGGATGATTTCCTGGGTGAAG |
| *pyrB* | Forward | ACCGTGGCGTGGAGATCGAG |
|  | Reverse | GCTGGCGTTGGGTGTTCTGG |
| *pyrC* | Forward | AACTCGGACTCTGGCGTGACC |
|  | Reverse | GTGCTCGTCGATGAACTGCTTCTC |
| *pyrE* | Forward | GCGGTGATCGACAGCGGAATC |
|  | Reverse | GTGGTCCTTGGCTTCCTTGCG |
| *pyrF* | Forward | CGACCTGAAATTCCACGACATCCC |
|  | Reverse | GTTGACCATCCACACGCCCATC |
| *luxS* | Forward | AAGTCCTATGGGTTGCCAAACTGG |
|  | Reverse | GCAGGCACTTCAGTAGCATTTAGC |
| *saeR* | Forward | CGCCTTAACTTTAGGTGCAGATGAC |
|  | Reverse | ACGCATAGGGACTTCGTGACCATT |
| *sigB* | Forward | GGTGCCATAAATAGATTCGATATGTCCTT |
|  | Reverse | CTTTTGATTTCACCGATTACAGTAGGTACT |
| *cidA* | Forward | AGCGTAATTTCGGAAGCAACATCCA |
|  | Reverse | CCCTTAGCCGGCAGTATTGTTGGTC |
| *icaA* | Forward | GTTGTCGACGTTGGCTACTG |
|  | Reverse | ATGGCAAGCGGTTCATACTT |
| *atlA* | Forward | AACAGCACCAACGGATTAC |
|  | Reverse | CATAGTCAGCATAGTTATTCATTG |
| *psmα* | Forward | GGCCATTCACATGGAATTCGTAG |
|  | Reverse | TAGCCATCGTTTTGTCCTCCTG |
| *psmβ* | Forward | GGACTAGCAGAAGCAATCGCA |
|  | Reverse | CCTAGTAAACCCACACCGTTAGC |
| *gyrB* | Forward | GGAGGTAAATTCGGAGGT |
|  | Reverse | CTTGATGATAAATCGTGCCA |

**Supplementary** **Table 2 Sequence of antisense oligonucleotides**

| Oligonucleotides | Sequence (5’-3’) |
| --- | --- |
| Oligo 1 | T*A*G*C*G*G*G*A*A*C*G*A*T*G*G*A*A*G*G*A |
| Oligo 2 | T*A*T*G*G*A*T*A*T*G*C*C*C*G*G*A*T*T*G*C |
| Oligo 3 | G*G*G*T*T*G*C*C*A*A*A*C*T*G*G*T*T*T*C*T |

**Supplementary** **Table 3 LP4C induced significant differently expressed genes**

| **GeneID** | **Name** | **log_2_FoldChange** | **Function** |
| --- | --- | --- | --- |
| RS05945 | *pyrF* | -3.23 | orotidine-5'-phosphate decarboxylase |
| RS05950 | *pyrE* | -3.11 | orotate phosphoribosyltransferase |
| RS05940 | *carB* | -2.85 | carbamoyl-phosphate synthase large subunit |
| RS05935 | *carA* | -2.31 | carbamoyl-phosphate synthase small subunit |
| RS05920 | *pyrP* | -1.89 | uracil permease |
| RS05925 | *pyrB* | -1.89 | aspartate carbamoyltransferase catalytic subunit |
| RS02315 | *TC* | -1.86 | neurotransmitter:Na+ symporter, NSS family |
| RS05930 | *pyrC* | -1.85 | dihydroorotase |
| RS05955 |  | -1.69 | Secretion system |
| RS05875 | *yggT* | -1.46 | YggT family protein |
| RS05905 | *lspA* | -1.21 | signal peptidase II |
| RS09440 | *crcB* | -1.11 | fluoride exporter |
| RS05915 | *upp* | -1.10 | pyrimidine operon attenuation protein /  uracil phosphoribosyltransferase |
| RS13915 | *rpfB* | -1.03 | resuscitation-promoting factor RpfB |
| RS12660 | *gltS* | -1.03 | glutamate:Na+ symporter, ESS family |
| RS01210 | *fadD* | -1.01 | long-chain acyl-CoA synthetase |
| RS10645 |  | 1.01 | arginine deiminase activity |
| RS10615 |  | 1.02 | serine-type endopeptidase activity |
| RS00800 | *adhE* | 1.11 | acetaldehyde dehydrogenase / alcohol dehydrogenase |
| RS14295 | *arcA* | 1.17 | arginine deiminase |
| RS10635 |  | 1.26 | Enzymes |
| RS10600 |  | 1.27 | orotidine-5'-phosphate decarboxylase activity |
| RS10605 |  | 1.30 | glutamate:sodium symporter activity |
| RS10610 |  | 1.34 | magnesium ion binding,  orotate phosphoribosyltransferase activity |
| RS10640 | *clpP* | 1.37 | ATP-dependent Clp protease, protease subunit |
